# Comparing Dual and Single‐Task Postural Performance in Older Adults with a Fall‐Related Chronic Mild Traumatic Brain Injury

**DOI:** 10.1002/alz.087953

**Published:** 2025-01-03

**Authors:** Amaya Sommer, Albert K Okrah, Stephen A Parada, Wanda D Jirau‐Rosaly, Max Bursey, Bradford Reynolds, Chijioke Ohamadike, Deborah A Jehu

**Affiliations:** ^1^ Augusta University, Augusta, GA USA

## Abstract

**Background:**

Fall‐related mild traumatic brain injuries (mTBI) are prevalent among older adults and are a predictor of dementia. Delays in diagnoses lead to prolonged symptoms and impairments. Dual‐task posture may be more sensitive to detecting impairments compared to single‐task posture, but research is limited. The objective of this study was to characterize single and dual‐task postural performance in older adults with a fall‐related chronic mTBI.

**Methods:**

We included community‐dwelling older adults who were seeking medical attention and/or still experiencing ongoing symptoms of a fall‐related chronic mTBI in this cross‐sectional study (n = 3; Age: 68.6 ± 8.1 years, Montreal Cognitive Assessment = 23 points, 67% Female). Dementia was an exclusion criterion. Participants stood on an AMTI force platform for 60 seconds and completed two repetitions of single‐tasks (standing with feet apart) and dual‐tasks (standing with feet apart while silently counting backwards by 3’s). Variables assessed were the 95% confidence area (m^2^), center of pressure (COP) path length (m), COP velocity (m/s), and mean frequency (Hz). A two‐way Condition (single, dual) by Measure (95% confidence area, COP path length, COP velocity, mean frequency) repeated measures analysis of variance was performed to compare single and dual‐task postural performance.

**Results:**

There was a significant Condition by Measure interaction (F_(3,6)_ = 11.53, p<0.01, η_p_^2^ = 0.85). Post hoc comparisons revealed a trend for greater path length (t_(2)_ = ‐3.38, p = 0.08; single‐task: 0.80 m, dual‐task = 0.84 m) and velocity (t_(2)_ = ‐3.38, p = 0.08; single‐task: 0.133 m/s, dual‐task = 0.139 m/s) during dual‐tasking compared to single‐tasking. No differences were shown in the 95% confidence area or mean frequency (p>0.05).

**Conclusion:**

The trend towards greater COP path length and COP velocity during dual tasking highlights that increased attention demand likely provokes postural instability. These measures may be sensitive to detecting impairments in dual‐task postural control; however, these findings will be confirmed as we enroll further participants in this ongoing study. Refined screening protocols for fall‐related mTBIs may the improve time to diagnosis, prevent reinjury, and mitigate long‐term impairments, including the potential onset of dementia. Future research is needed to determine if cognitive impairment is a risk factor for fall‐related mTBIs in older adults, as all our participants had mild cognitive impairment.

